# Examining the sexual function and related attitudes among aged women: A cross- sectional study

**Published:** 2016-01

**Authors:** Safieh Jamali, Afifeh Rahmanian, Shohreh Javadpour

**Affiliations:** 1 *Research Center for Social Determinants of Health, Jahrom University of Medical Sciences, Jahrom, Iran.*; 2 *Department of Nursing, Faculty of Nursing and Midwifery, Jahrom University of Medical Sciences, Jahrom, Iran.*

**Keywords:** *Sexual dysfunction*, *Menopause*, *Women*, *Attitude toward sexuality*

## Abstract

**Background:**

Sexual function and its subsequent satisfaction are among the most important aspects of women’s life. However, this instinct could be influenced by some factors such as diseases, drug using, aging, and hormonal and physiologic changes associated with menopause, and sexual behavior.

**Objective:**

The aim of this study was to describe the prevalence rates of sexual dysfunction, and related attitudes among aged women in Jahrom, Iran.

**Materials and Methods:**

This cross-sectional study was conducted on 746 postmenopausal women aged between 50 and 89 years old who had referred to obstetric and gynecologic clinic in Jahrom, from April to October 2014. Female Sexual Function Index questionnaire was used order to assess the sexual function. The cases were classified into three categories according to the attitude scores: negative (17-32), medium (33-38), and positive (39-48). One-way ANOVA test was used to determine the relationship between FSFI and attitude scores.

**Results:**

The participants’ mean±SD age was 60.10±6.89 years and the total mean score of FSFI was 19.31±8.5. In addition, 81.5% of the women had sexual dysfunction (FSFI< 26.55) and only 147 women (18.5%) had normal sexual function (FSFI> 26.55). Almost 62.1% the women displayed a negative attitude towards sexuality and only 18.8% women had positive attitude. Feeling of dyspareunia (p= 0.02), lubrication (p< 0.0001), orgasm (p= 0.002) and satisfaction (p= 0.002) were significantly different between three categories of attitudes regarding sexuality, respectively

**Conclusion:**

Our data showed that sexual disorders were highly prevalent among postmenopausal women. The most affected problems were arousal, dyspareunia, and lubrication. More than half of the women had negative attitude towards sexual function consequently this could affect their sexual function. So, it seems screening of sexual dysfunction for finding the causes in women should be the main sexual health program. Also, it would be important to emphasis the role of physicians and experts on education and counseling in this subject.

## Introduction

Sexual relationship is a complex phenomenon influenced by cardiovascular, nervous and hormonal factors, as well as individuals' biological characteristics, inter-personal relationships, established traditions in families and societies, cultures and religions (1). Sexual disorders in women are often defined as permanent or chronic disorders in one or more of the following four areas: sexual desire, sexual arousal, painful intercourse, and failure to experience orgasm (1, 2). 

Menopause is an important time for middle-aged women, with personal, cultural and social consequences, and is treated as a major health issue in women's healthcare (2). The sexual response cycle in women is done by complex interactions, psychological, social, environmental and biologic factors (hormonal, vascular and neuro-muscular) (3). Although increasing the age is the main factor of decreased sexual function in elderly women, but hormonal changes could be important in sexual function. Menopause affects the sexual desire because of physiological and psychological changes (4).

Initial biological changes include reduction of estrogen level. Initial estrogen deficit results in irregular menstruation and low vaginal lubrication. Constant loss of estrogen associated with vascular, muscular, genito-urinary, sleep and mental changes result in direct and indirect effects on sexual function. Also, decrease of testosterone and androgen function related to age facilitate and the women sexual dysfunction (5).

This evolutionary process results in a cessation of periods, reduction in the activity of the ovaries, and changes in hormone levels, which in turn lead to a variety of physical, emotional, psychological and social complications (6). On the other hand, sexual activity is a major issue in individuals’ quality of life. Increase of life expectancy and the growing elderly population have made sexual health as an important issue in menopausal women (7). 

Though certain physiologic and pathologic changes are responsible for the aforementioned complications during menopause, women's attitude plays an important role in causing or eliminating problems (8, 9). Some women consider menopause as the beginning of their freedom, as they will not worry about pregnancy and suffer the problems of menstruation anymore, and thus may appear to be more sexually active than before. However, other women deem this time as heralding old age and loss of their attractions and consequently had less sexual function (10-12).

Researchers demonstrated that 35% of menopausal women reported reduction of sexual desire and 62% reported this disorder in various stages of life. The prevalence of reduction of sexual desire was reported to be 47%, 54%, 42%, and 24% in English, Italian, French, and German menopausal women, respectively (13).

Lumen* et al *showed that the menopausal period as a major factor in increasing sexual disorders in women (14). Other studies report the prevalence of women sexual dysfunction 25-65% in the society and this will be increased to 68-86% in geriatrics (15). A study conducted on 163 married women of 18-65 years old revealed that the prevalence of women sexual dysfunction is 25.8% and the most common disorder is the desire disorder (16). Arman *et al *showed that the prevalence of women pre-menopausal sexual dysfunction is 38.5% and is increased to 72.4% (17). The study of Sheykhan *et al* conducted on 46-50 years old women in Tehran showed that 42% of women had unfavorable sexual function (18). Bonnie and Saks reported that the incidence of sexual disorders in the menopausal period is 20%, while Castelo declared it to 50% and it increased with age (19, 20). 

Different statistical prevalence of women sexual dysfunction in studies carried out across the country is associated with different customs and culture of women. Because Iran is a multicultural country and according to socio-cultural and ethical factors affecting sexual function in menopause, it is necessary to study on this subject in other part of Iran. Besides, the best questionnaire to assess women sexual dysfunction in the menopause period is Femal Sexual Function Index (FSFI) that is used in less Iranian studies (21). 

Most of studies in Iran assess the prevalence of women sexual dysfunction and related factors in menopause and studies have done to determine the attitude of women about the sexual function in the menopausal period since the attitude of post menopausal women towards sexual activity affects the sexual function (22). In the other hand, the social and cultural barriers, taboos and misunderstandings, race differences, ethnicity, culture and traditions of the society are factors related to different prevalence of sexual dysfunction among the countries (23). It seems that there are false attitudes and unbalanced social traditions towards sexual matters in this era. So, it was decided to study the incidence of sexual disorders and their relationship with the attitude to sexuality in post menopause Iranian women.

## Materials and methods

The present cross-sectional study was conducted on 746 postmenopausal women referring to obstetric and gynecologic clinic in Jahrom, from April to October 2014. The study samples were selected among the menopause women referring to the clinic through convenience sampling. Two midwifery specialists trained the study subjects about how to complete the questionnaires and after signing written informed consents.

The inclusion criteria were Menopausal women who have passed their 12 months of amenorrhea and ≥ 50-years-old. All women were lived with their sexual partners and had a regular sexual partner in the previous 4 weeks. Additionally, women should have an intact uterus and both ovaries. First, medical history was taken from women who had tendency to participate in this study. Medical history was consisted of medical disease (blood hypertension, diabetes mellitus, hyperlipidemia), positive history of surgery (hysterectomy, mastectomy, cystocele, rectocele, oophorectomy) and drugs (cardiac medication, diabetes medication, antipsychotic drugs). 

Women with positive aforementioned history, were prohibited to participate in the research. Among the 864 cases, 746 completed the questionnaires and 46 were unwilling to take part in the study. The other exclusion criteria were smoking and drug abusing, women with continuous hormone therapy, having physical problems of spinal cord injury, mutilation, paralysis, having psychological problems or taking antidepressant or sedative medications, and those who were unwilling to participate in the present study. Based on exclusion criteria, 72 women were excluded from the study because of the positive history. Regarding the ethical considerations, the questionnaires were completed anonymously. 

Besides, after explaining the study objectives, written informed consents were obtained from all of participants. The researchers tried to gain the participants’ trust by creating good relationships, performing interviews at appropriate time and place, and providing the necessary information about the research objectives. The study was approved by the Ethics Committee of the Jahrom University of Medical Sciences (REC.1392.045.JUMS). 

Data collection tools were questionnaires. These questionnaires were composed of three parts; the first part was the demographic part, which was included age, education, occupation). The second part was to assess sexual function by FSFI. The third part was the sexual attitude by self-made questionnaire. FSFI questionnaire was introduced by Rosen *et al* (24). This questionnaire consists of 19 questions investigating the subjects in 6 domains of sexual desire, sexual arousal, lubrication, orgasm, sexual satisfaction, and pain during intercourse. 

In this questionnaire, the questions are scored based on 0 or 1-5 scoring system and the score of each domain is calculated through summing up the scores of that domain’s questions and multiplying the obtained number by the multiplier factor of that domain. It should be mentioned that sexual desire is covered by questions 1 and 2, excitement by the sum of questions 2, 4, 5, and 6, lubrication by adding questions 7, 8, 9, and 10, orgasm by the sum of questions 11, 12, and 13, sexual satisfaction by adding questions 14, 15, and 16, and pain by summing up questions 17, 18, and 19. In addition, multiplier factors of 0.6, 0.4, and 0.3 are used for domains including 2, 3, and 4 questions, respectively. In general, each domain has a minimum (0-1.2/1.8) and a maximum (6). 

In addition, the sexual function total score is obtained from the sum of the scores of all the domains and is ranged from 2 to 36. The cut-off point of 26.5 was used for determining the sexual dysfunction; in a way that FSFI<26.5 was considered as suffering from sexual dysfunction and FSFI≥ 26.5 was considered as having normal sexual function. The cut off scores to determine the presence of difficulties on the six domains of the FSFI were obtained from previous studies (24-26). Accordingly, scores less than 4.28 on the desire domain, less than 5.08 on the arousal domain, less than 5.45 on the lubrication domain, less than 5.05 on the orgasm domain; less than 5.04 on the satisfaction domain, and less than 5.51 on the pain domain were used to classify participants as having difficulties in that domain. Researchers translated this questionnaire to the language of their study populations and determined its reliability and validity, as well. 

Overall, FSFI questionnaire is a general standard one whose reliability and validity were determined by Rosen *et al* (27). Mohammadi also performed a study in Iran in 2004 and confirmed the reliability as well as the validity of the questionnaire (28). The Persian version of FSFI was used because Persian is the main national language in Iran. Sexual attitudes questionnaire were included 12 questions that required respondents to indicate whether they agree or disagree with the statements using the following scales: 1- disagree, 2- undecided, 3- agree. A final score was obtained for the total scale by summing responses graded with scores, ranging between 12-48. 

Lower scores represented negative sexual attitudes while higher scores showed positive attitudes. They were divided into three categories: negative (scores 17-32), medium (scores 33-38), and positive (scores 39-48).questionnaire's face and content validity were evaluated by 10 gynecologists and psychiatrists who were expert in the field of sexual health. The questionnaires were changed based on their comments. The reliability of the questionnaire was assessed using test- retest and inter- rater method. In these methods, first a trained questioner completed questionnaires for 30 participants, then another observer filled the same questionnaire. 

The results of two observers were compared using statistical analysis. The inter- rater reliability and test- retest reliability were confirmed by r= 0.91 and r= 0.85 respectively (29, 30).

**Statistical analysis**

Finally, the data were analyzed statistically with Version 16.0. Chicago, SPSS Inc) and descriptive statistics (including frequency, percent, mean, standard deviation, maximum and minimum) were used to present the socio-demographic variables. One- way ANOVA tests were used to determine the inter-domain correlations in the groups. Besides, p< 0.05 was considered as statistically significant.

## Results

The age range was 50-89 and with the mean age of 60.10±6.89 years. Almost 36.7% of the cases had no formal education. Most of the women (40.6%) in our study group were housewives. Among the women who answered the question about body mass index (BMI), most (42.5%) were obese. Of all, only 35 women (4.7%) were current smokers. Almost 60.5% of women had no knowledge on menopause (Table I).

**Prevalence of Female Sexual Dysfunction**

Comparing the sexual function in each domain, the lowest mean score was noted in the domain of desire (2.82±1.40), arousal (3.10±1.55), followed by orgasm (3.11±1.73), pain (3.25±1.73), lubrication (3.31±1.78), and satisfaction (3.72±1.50). Domain scores suggestive of difficulties related to desire, arousal, lubrication, orgasm, poor satisfaction, and pain were prevalent in 647 (86.7%), 682 (91.8), 659 (88.6%), 646 (86.9%), 593 (79.7%) and 672 (90.40%) subjects, respectively. The prevalence of the sexual problems is shown in table II.

About 22.7% of postmenopausal women reported “never or almost never” feeling of sexual desire. Thirteen percent of postmenopausal women reported about their arousal problems as “never or almost never” experiencing arousal during sexual activity. 64 of the postmenopausal women had not experienced orgasm during intercourse. In addition, 21.6% had no lubrication during sexual activity. Inter-domain correlations were statistically significant and ranged from r=0.52 to r=0.91. The highest correlations were between orgasm and lubrication (r=0.83), orgasm and arousal (r=0.80), as shown in table III.

**Attitudes of the participants towards sexuality**

Respectively, 62.2, 19.2, and 18.8% of women had negative, moderate, and positive attitude towards sexual function after the menopause. A survey of women's perception of sex after the menopause revealed that 79.2% believed that sex is forbidden for menopausal women according to religious creeds. 

However, 6.2% believed that maintaining a sexual relationship with their husbands after the menopause would keep their husbands satisfied, 64.2% stated that sex after the menopause is embarrassing. 

66.2% believed that sex after the menopause is unacceptable in the Iranian culture; also, 67% stated that due to the physical changes accompanied with ageing, they were too embarrassed to maintain their sexual relationship with their husbands, and only 30.6% found sex after the menopause attractive because they did not worry about becoming pregnant (Table IV). 

There was not a statistically significant relationship between sexual desire and sexual arousal on one hand and sexual attitude on the other hand. However, the other aspects were correlated with lubrication, orgasm, dyspareunia, satisfaction and the total sexual function scores (p<0.05) (Table V).

**Table I:** Participants' characteristics (n=746)

**Characteristics**	**Mean±SD**
Age	60.10±6.89
Duration of menopause	8.33±6.11
Age of menopause	50.19±1.92
	**N (%)**
Age	
50-55	242 (32.4)
56-60	166 (22.3)
> 60	338 (45.3)
Educational level	
Uneducated	274 (36.7)
Primary school	201 (26.9)
Secondary school	145 (19.6)
College or University	125 (16.8)
Employment status	
Housewife	303 (40.5)
Employed	183 (24.5)
Retired	260 (34.9)
Smokers	
Yes	35 (4.7)
No	711 (95.3)
Body Mass Index (BMI)	
BMI 20-24.9	177 (23.7)
BMI 25-29.9	252 (33.8)
BMI >29.9	317 (42.5)
Any knowledge on menopause?	
Yes	451 (60.5)
No	295 (39.5)
The source of information on menopause	
Physician, Midwife/nurse	268 (35.9)
Neighbors-Relatives	387 (51.9)
Books-Magazines-Newspaper-TV-radio-internet	91 (13.2)

**Table II:** Prevalence of sexual dysfunction according to female sexual function index scores among women (n= 746)

**Domain**	**Sexual dysfunction**		**No sexual dysfunction**		**No sexual dysfunction**
	**n**	**%**	**n**	**%**	
Desire	647	86.7	99	13.3	2.82 ± 1.40
Arousal	682	91.8	61	8.20	3.10 ± 1.55
Lubrication	659	88.6	85	11.40	3.31 ± 1.78
Orgasm	646	86.9	97	13.10	3.11 ± 1.73
Satisfaction	593	79.7	151	20.30	3.72 ± 1.50
Pain	672	90.40	71	9.60	3.25 ± 1.73
Total Score	599	81.5	136	18.50	19.31 ± 8.50

**Table III:** Inter-domain correlations for female sexual function index (FSFI) total score and domain scores (n= 746)

**Domain**	**Desire**	**Arousal**	**Lubrication**	**Orgasm**	**Satisfaction**	**Pain**	**Total score**
Desire	1						
Arousal	0.72	1					
Lubrication	0.67	0.77	1				
Orgasm	0.66	0.80	0.83	1			
Satisfaction	0.68	0.72	0.79	0.77	1		
Pain	0.52	0.67	0.68	0.69	0.61	1	
Total Score FSFI	0.80	0.89	0.91	0.91	0.86	0.68	1

**Table IV:** The attitude towards sexual activity in the postmenopausal period

	**% of ** **dis** **agree**	**% of ** **agree**	**% of ** **uncertain**
Having sexual activity is natural normal thing in menopausal period	66	27.7	6.3
Having sexual activity makes the postmenopausal women happy	34.3	45.2	20.5
Having sexual activity makes the postmenopausal women’s partners happy	82.8	6.2	11
No sexual activity affects the postmenopausal women’s life so much	26.8	65.1	8
Having sexual activity in menopause is the embarrassing thing because of old age	31.6	64.2	4.2
Having sexual activity in menopause is very shy in Iranian society	32.6	63.3	4.2
Having sexual activity in menopause is the bad behavior and oppose to Iranian culture	30.7	66.2	3.1
Having sexual activity in menopause is prohibited from religious beliefs	17.8	79.2	2.9
You are very nervous and shy if others know that you still have sexual activity	31.9	65.1	2.9
Postmenopausal women should go to the temple and make the merit instead of thinking about sexual activity	17.4	60.3	22.3
Their body images are changed and make them too embarrassed to have sexual activity with their partners	30	67	3
Having sexual activity during the menopause is very happy because they don’t concern about getting pregnancy	64.2	30.6	5.2

**Table V:** Female Sexual Function Index scores according to* attitude towards sexuality* in women

**Domain**	**Negative (n= 463)**	**Medium (n= 143)**	**Positive (n= 140)**	**p-value** *	**Total (** **n= 746)**
Desire	2.76 ± 1.44	2.92 ± 1.25	2.92 ± 1.40	0.31	2.82 ± 1.40
Arousal	3.02 ± 1.63	3.14 ± 1.44	3.31 ± 1.34	0.15	3.10 ± 15
Lubrication	3.12 ± 1.82	3.54 ± 1.80	3.72 ± 1.55	0.0001	3.31 ± 1.78
Orgasm	2.95 ± 1.77	3.2 ± 1.72	3.54 ± 1.54	0.002	3.11 ± 1.73
Satisfaction	3.58 ± 1.49	3.87 ± 1.49	4.06 ± 1.51	0.002	3.72 ± 1.50
Pain	3.17 ± 1.79	3.15 ± 1.63	3.62 ± 1.61	0.021	3.25 ± 1.73
Total Score FSFI	18.57 ± 8.82	19.79 ± 7.89	21.22 ± 7.46	0.004	19.30 ± 8.46

* P-value one way ANOVA test between first, second, and third group

## Discussion

The results of the study showed that the frequency of sexual dysfunction in post-menopausal women is 81%. According to the study of Krantarat *et al*, 82.2 % of menopausal women suffer from sexual dysfunction (30). According to another study performed in the U.S.A., 50% of women aged between 57-85 are affected by sexual dysfunction (31). According to the study of Omidvar performed in Amol, Iran, 54% of menopausal women suffer from sexual dysfunction (32). However, Arman *et al* reported the prevalence of post- menopausal sexual dysfunction in Isfahan, Iran, is 72% (17). The study of Hashemi *et al* showed that two third of 45-65 years- old women suffer from at least one sexual problem (29). Also, the study of Nazarpour *et al* on 405 women aged of 40-65 years old in Chalus, Iran, revealed that 61% of post-menopausal women had sexual dysfunction (4). The high prevalence of sexual dysfunction among menopausal women in Iran can be attributed to their attitude: 62.2% of the interviewed women had a negative attitude to sexuality after the menopause. Moreover, the results showed that there was a significant relationship between women's sexual function and their attitude toward it (p= 0.004): women with a negative attitude had a lower sexual function mean score. Thus, it can be concluded that women's sexual function is deeply influenced by their attitude. Social attitudes and cultural roles and religious beliefs can affect older women’s experience of sexual desire (33).

Nisar and Ahmed-Sohoo mentioned that post-menopausal women from traditionalsocieties often tend to take care of their children and grandchildren and to perform religious ritual, rather than participation in sexual activities is the next priority (34). da Silva Lara and colleagues, determined in their review that 22% of post-menopausal women participated in sexual activity just to satisfy their spouses and had no desire to participate in these activities (10). Menopause is a complex biological phenomenon by physiological and socio- cultural factors that leads women have various attitudes towards it (35). The attitude with respect to community and culture to make a difference is the frequency of sexual dysfunction in both internal and external studies. The results of the present study show, 91% of women suffer from arousal disorders. In a similar study by Kabudi in Iran, 70% of menopausal women were suffered from sexual arousal disorders; Omidvar *et al* found it to be 80%, and Arman *et al* reported a 75% rate of occurrence (17, 32, 36). 

As with the present study, all three above studies declared sexual arousal disorders to be the most prevalent type of sexual-function-related disorders. However, Olaoloram and Lawoyin, in Nigeria, and Valadares et al found the prevalence of sexual arousal disorders to be 40 and 35.9%, respectively (37, 38). Frequent problems associated with this phase include vaginal dryness and dyspareunia (painful intercourse) (39). The results generated from this study showed that there is a positive and significant relationship between the arousal stage and lubrication (p<0.0001, r=0.77) dyspareunia (p<0.0001, r=0.67), which is an indication of the interaction among sexual phases.

90% of the women in the present study were suffered from dyspareunia. However, Omidvar *et al* and Hashemi *et al* (29, 32). Reported a prevalence 55%. Studies in Australia, Taiwan, and Turkey show the prevalence of dyspareunia to be, respectively, 12, 32, and 16% (40-42). On the other hand, 88.6% of the participants weresuffered from vaginal dryness. Omidvar *et al* declared the prevalence of the disorder to be 80%, which is in agreement with the present study (32). However, in Thailand and Taiwan, this prevalence is reported 20 and 23.6% respectively (43, 41). Some researchers reported that menopause affect the attitude and sexual activity of women, but the study of Sheykhan *et al* showed that low sexual activity simultaneously increases the age that is more related to culture and behavior of people than human physiology or hormones (18, 44, 45). Avis *et al* stated that transition to menopause has fewer effects on sexual function than relationship variables, cultural environment, and attitudes regarding sex (46). On the other hand, some studies mentioned the role of hormones in sexual response (47). It was shown that titer of serum testosterone in post- menopausal women is associated with sexual function (48, 13, 49). Also, low sexual desire, arousal and lubrication are related to low androgen level (50). Some studies revealed that intrinsic or synthetic androgen, potentially affects the sexual function (51).

The study of Hashemi *et al* performed in Tehran, Iran, showed that disorder of sexual desire or orgasm in post menopausal women is associated with negative attitude of women towards sexuality (29). Our data showed that there was a significant relationship between negative attitude and pain, lubrication, orgasm and satisfaction. Also, there was a significant relationship between the participants' attitude and the disorders related to dyspareunia (p=0.021), lubrication (p<0.0001), and orgasm (0.002). According to our results, 86% of the participants were suffered from orgasm disorders. However, Omidvar *et al* and Nicol *et al* reported the prevalence was 25 and 16%, respectively (32, 52). Advanced age, emotional and psychological disorders, medication and diseases can adversely affect women's sexual satisfaction. 

Vaginal dryness and dyspareunia can also prevent women from reaching orgasm (53). The results of the present study showed that the negative attitude of menopausal women to sex is correlated with a sharp decline in experiencing orgasm (p=0.002). The impact of women's sexual attitude on the other aspects of their sexual satisfaction (p=0.002) and total sexual function score (p= 0.004) is definite. The women with a negative attitude obtained lower satisfaction scores than the others. The majority of the participants stated that having sex at their age is embarrassing and is against the Iranian culture. 6.2% believed that having sex after the menopause would keep their husbands satisfied, and 67%, due to the physical changes that appear with age, found sex after the menopause shameful. 

Krantarat *et al,* however, reported 2.3% prevalence of a negative perception of one's body during and after the menopause (30). Omidvar *et al* showed that 52.5% of Iranian women believe that sexual dysfunction adversely influences their relationships with their husbands (32). The study of Nicolos *et al* showed that 76% of women see sexual satisfaction as integral to a relationship (52). It can be concluded that sexual dysfunction is influenced by such factors as ethnic, religious, cultural, and attitude matters. Moreover, it appears that the high prevalence of sexual disorders in the present study are related to the history and unresolved sexual problems of the participants during their fertile years, only to be aggravated by the menopause. 

**Strengths and Limitations of the Study**

One of the strong points of this study was using FSFI questionnaire, which contains all the key dimensions of sexual function, and has a high reliability as well as validity, that has been less used in Iranian population. In addition, the individuals referring to clinic had a specific socio-cultural status and a considerable amount of time had to be spent for explaining the questions and obtaining accurate answers. Nevertheless, since it was the first time that these issues were discussed with them, their answers were quite honest and reliable.

The limitation of our research is that this study was performed in Iran for the first time and the result of the study from Jahrom (a city of Iran) cannot be generalized. One of the limitations of the study is lack of measuring the participants' emotional-psychological growth, which is an important factor in a couple's relationship and satisfaction. Moreover, since one's sexual relationship is a highly private matter and there are cultural and religious taboos surrounding it, it is possible that some women are not comfortable about discussing their sexual lives. The probable reticence of some of the participants about their sexual activities was a limitation that the researcher could not control. The population of this study was limited to women referred to obstetric and gynecologic clinic only, and the findings may not be generalizable to the entire population. The weakness of our study is that sexual function of the partners did not assess, as regards male sexual dysfunction can be one of the effective factors on couples’ sexual function.

## Conclusion

The results showed that sexual disorders are prevalent among women during their menopausal years, and such disorders have a positive and significant relationship with women's attitude to sex. Screening menopausal women for sexual dysfunction should become a health-care priority. It is also recommended that menopausal women be educated on sexual function, the physiological changes of menopause period, and how to adapt to them. Also, the entire health- care personnel who work with the elderly women should take care to send the patients who are suffering from such disorders to psychologists for advice. More researches on sexual dysfunction before and after the menopause should be performed for better conclusion. Also, more in-depth qualitative studies should be conducted to determine women's sexual behavior and attitude to sex. 
